# Letter to the editor

**DOI:** 10.1186/2051-1426-2-22

**Published:** 2014-07-15

**Authors:** Camilo E Fadul, Jan L Fisher, Thomas H Hampton, Marc S Ernstoff

**Affiliations:** 1 Geisel School of Medicine at Dartmouth, Lebanon, USA; 2 Dartmouth-Hitchcock Medical Center, Lebanon, USA; 3 Norris Cotton Cancer Center, One Medical Center Drive, Lebanon NH 03756, USA

## 

May 8, 2014

Pedro Romero, M.D.

Editor-in-Chief

Journal for ImmunoTherapy of Cancer

Dear Dr. Romero,

We read with interest the work by Olin and collaborators published in the *Journal for ImmunoTherapy of Cancer* under the title “Vaccination with dendritic cells loaded with allogeneic brain tumor cells for recurrent malignant brain tumors induces a CD4 + IL17+ response” [1]. Immune therapy trials for malignant brain tumors are challenging to undertake and there are many factors, including the high expense per patient which limits the number of patients enrolled, hence curtailing the ability to demonstrate that the intervention improves clinical outcomes. Furthermore, the baseline immune competence of patients with gliomas is heterogeneous and is an important variable in determining who will benefit from this therapeutic modality. Assessment of immune markers before treatment with immunotherapy which could reliably predict response is highly desirable, since patients unlikely to benefit could be considered for alternate clinical trials.

Profiling the immune cell populations in peripheral blood by flow cytometry is a potentially useful tool, and hierarchical clustering analysis of the multiple cell populations measured may provide a distinct and comprehensive overview of the immune status [2]. Olin et al. use this methodology to separate patients with brain tumors who had stable or progressive disease after being treated with dendritic cells loaded with allogeneic tumor. We would like to bring to the attention of the authors and readership that in our 2011 publication, we applied hierarchical clustering to functional immune assay measures of antigen specific response to treatment with autologous tumor lysate loaded DC vaccination in a cohort of glioblastoma patients [3]. The shift of immune response measures discerned 2 groups of patients that correlated with survival.

Successful use of hierarchical clustering analysis of multiple immune measures as a prognostic and predictive marker in patients with gliomas first reported by us and confirmed by Olin et al., serves as a proof of principle. A substantially larger study using supervised classification techniques to identify the key prognostic factors, will allow validation of a prediction system. Integrated biomarkers that are able to more accurately and reliably quantify immune competence are needed to be able to do clinical trials enriched for individuals likely to benefit from immune therapy.

Sincerely,

Camilo E. Fadul, MD

Jan L. Fisher, MS

Thomas H. Hampton, MS

Marc S. Ernstoff, MD

## Competing interests

The authors declare that they have no competing interests.

## Authors’ contributions

All authors drafted, read and approved this letter. CEF, JLF, THH, MSE.

## References

[B1] Olin M Low W McKenna D Haines S Dahlheimer T Nascene D Gustafson M Dietz A Clark H Chen W Blazar B Ohlfest J Moertel C Vaccination with dendritic cells loaded with allogeneic brain tumor cells for recurrent malignant brain tumors induces a CD4+IL17+ response J Immuno Ther Cancer 2014 2 1 4 PubMed PMID: doi:10.1186/2051-1426-2-4 10.1186/2051-1426-2-4 PMC4019901 24829761

[B2] Gustafson M Lin Y LaPlant B Liwski C Maas M League S Immune monitoring using the predictive power of immune profiles J Immuno Ther Cancer 2013 1 7 18 PubMed PMID: doi:10.1186/2051-1426-1-7 10.1186/2051-1426-1-7 PMC4266565 25512872

[B3] Fadul CE Fisher JL Hampton TH Lallana EC Li Z Gui J Szczepiorkowski ZM Tosteson TD Rhodes CH Wishart HA Lewis LD Ernstoff MS Immune response in patients with newly diagnosed glioblastoma multiforme treated with intranodal autologous tumor lysate-dendritic cell vaccination after radiation chemotherapy J Immunother 2011 May 34 4 382 389 PubMed PMID: 21499132. Pubmed Central PMCID: 3766324 10.1097/CJI.0b013e318215e300 21499132 PMC3766324

